# Knowledge exchange in crisis settings: A scoping review

**DOI:** 10.1371/journal.pone.0282080

**Published:** 2023-02-24

**Authors:** Elizabeth McGill, Emma Halliday, Matthew Egan, Jennie Popay

**Affiliations:** 1 Department of Health Services Research and Policy, London School of Hygiene and Tropical Medicine, London, United Kingdom; 2 Faculty of Health & Medicine, Division of Health Research, Lancaster University, Lancaster, United Kingdom; 3 Department of Public Health, Environments and Society, London School of Hygiene and Tropical Medicine, London, United Kingdom; University of Vermont, UNITED STATES

## Abstract

**Background:**

Public health practice and efforts to improve the social determinants of health operate within a climate characterised by multiple and intersecting crises. This includes the Covid-19 pandemic as well as more protracted crises such as climate change and persistent social inequalities that impact health. We sought to understand and compare how knowledge exchange (KE) processes occur across different crises, and how knowledge on improving social determinants of health can be utilised at times of crisis to reduce health inequalities and strengthen public systems.

**Methods:**

We conducted a scoping review to understand how KE on improving social determinants of health can occur across different types of crises (e.g. environmental, pandemics, humanitarian). Relevant studies were identified through electronic searching of Medline, EMBASE, Global Health, Scopus and Web of Science databases.

**Results:**

We identified 86 studies for inclusion in the review. Most studies concerned pandemic or environmental crises. Fewer studies explored KE during technical (e.g. nuclear), terror-related or humanitarian crises. This may reflect a limitation of the searches. Few studies assessed KE as part of longer-term responses to social and economic impacts of crises, with studies more likely to focus on immediate response or early recovery stages. Exchange of research evidence or data with policy or practice contextual knowledge was common but there was variation in the extent that lay (public) knowledge was included as part of KE processes.

**Conclusion:**

As ongoing crises continue with significant public health implications, KE processes should appropriately reflect the complexity inherent in crises and foreground health inequalities. Doing so could include the utilisation of systems or complexity-informed methods to support planning and evaluation of KE, a greater focus on KE to support action to address social determinants of health, and the inclusion of a plurality of knowledge–including lived experience–in planning and responding to crises.

## Introduction

Public health practice and efforts to improve the social determinants of health–the social, environmental, political, economic factors shaping inequalities in health–currently operate within a climate characterised by multiple and intersecting crises. This context includes immediate public health emergencies such as the Covid-19 pandemic to more protracted crises, such as climate change and growing social inequalities and their impacts on health.

International attention to the social determinants in public health policy, practice and research, particularly in the global North, is not new, influenced by developments such as the World Health Organisation Commission on Social Determinants of Health [1]. However, the pandemic’s wide-ranging impacts also exemplifies how crises exacerbate health inequalities [2,3]. Recent research has found, for example, that individuals living in poverty or working in particular sectors were at higher risk of mortality from Covid-19 at a county level in the United States [4]. Indirect effects relate to the socio-economic consequences of crises (e.g. rising unemployment) which may be more adversely felt among particular groups in the population [5,6]. Beyond pandemics, other types of crises (e.g., conflict, environmental) may also have an immediate and direct impact on local population’s living and working conditions. For example, the destruction of housing and infrastructure can lead to the displacement of populations and the further exacerbation of inequalities [7,8].

Added to the public health challenge is the complex nature of crises. While crises can be conceptualised as existing in different stages, these stages frequently overlap and intersect with each other (see Table 1). As recent events exemplify (e.g., war, the cost of living crisis and the ongoing pandemic), such crises also rarely happen in isolation. Rather, they interact with broader system changes and adaptations [9–11]. In the UK, these broader system changes include Brexit and political and cultural movements such as Black Lives Matter and Me Too. Added to this, these intersecting crises and series of systems changes are occurring within a period of prolonged disinvestment in public services. A significant challenge, therefore, is to address the social determinants of health in order to reduce widening health inequalities [12,13] at a time when economic resources are constrained [14–16]. These efforts have implications for the type of knowledges that public health requires to respond and mitigate against the impact of crises.

**Table 1:** Crisis stages.

Crisis Stage	Definition	Examples of activities
Mitigation	Efforts to prevent or minimise the risks, hazards and anticipated damage stemming from a crisis; may occur before, during or after a crisis	Public health control measures; vulnerability analyses
Preparedness	Building capacity–physical, technological, resourcing–and plans to improve the ability to respond to a crisis; happens prior to a crisis	Development of surveillance, warning and reporting systems; emergency response planning and exercises
Response	Efforts to meet basic needs, minimise hazards and control crisis	Emergency relief; collection and analysis of epidemiological data; development and dissemination of best practice guidance; coordination and implementation of control measures
Recovery	Efforts to return to normal or adapt to ‘new normal’ conditions; occurs during and after the immediate crisis response	Monitoring long-term impacts; transition to normal service functions; rebuilding and strengthening systems

References: [9–11].

Within this context, there is a need to understand how KE processes occur across crises and how a range of different knowledges can be utilised to reduce health inequalities and strengthen public systems within an uncertain future. In particular, given that communities, practitioners, researchers and policy makers are tasked with responding to multiple crises, there is an opportunity to synthesise learning about models of KE across crises settings and the factors influencing its success in diverse contexts.

Knowledge exchange as a discipline has emerged particularly in the last four decades [17]. While effective KE is a critical mechanism to improve population health [12] and address health inequities [13], there are a range of concepts related to KE used (often interchangeably) to denote different dimensions, processes and the inclusion of different sources of knowledge [12,14,15]. Fazey and colleagues point to at least ten different concepts used to define knowledge exchange processes [18]. These terms include, for example, knowledge management, knowledge translation, knowledge transfer and knowledge exchange. The term knowledge management has often been utilised within an organisational context and generally refers to the systems and tools used to effectively store, manage and search relevant knowledge [16]. Other concepts are used to present the process in different ways in terms of the direction of the flow of knowledge. Knowledge transfer is generally defined as a one-way process in which research evidence is shared with relevant stakeholders, with limited attempt to modify or adapt the knowledge to a local context [12,14,17]. Knowledge translation is often also a one-way process but one in which research findings may be further interpreted, synthesised and presented in a format that makes them more useful to potential users of that knowledge. For example, some knowledge translation efforts may include adapting knowledge to suit different contexts [12,14,17]. Knowledge exchange implies a more collaborative, two-way or multi-dimensional or multi-disciplinary process whereby multiple and diverse sources of knowledge, including ideas, beliefs, evidence and expertise of a range of groups (e.g. publics, policy makers, practitioners) are actively shared with the goal of engaging in mutual learning [14,15,17,18]. KE processes are complex: sharing knowledge can involve the interaction of a range of different stakeholders and new understandings and practices may (but may not) emerge from these interactions [18–20].

As Fazey also describes, however, the use of different terms or processes go beyond technical differences, and reflect a more fundamental epistemological position on how knowledge is understood and valued [18]. Within a public health field shaped by a positivist paradigm, this has often meant certain forms of knowledges have been privileged, notably research evidence and the experiential knowledge of practitioners and policy makers [19,20]. In the context of action to tackle health inequalities, this has led to criticisms lay knowledge has not been privileged in understanding the causes and action to be taken [21,22]. Researchers putting forward such criticism have suggested that decision making spaces should also ‘*prioritis[e] listening to*, *and working to understand*, *the experiences of communities experiencing the brunt of health inequalities’* [23 p.268].

During crises more specifically, there are likely to be particular considerations affecting the success of KE that may vary across different crisis types and stages. Comparing KE conducted in different contexts may therefore facilitate learning and the transfer of knowledge about effective KE processes and mechanisms. Within the disaster management field, a growing body of evidence on KE exists in relation to pandemic124s, environmental, humanitarian, terror related and technical (e.g. industrial) disasters that has drawn out features of KE across different crises. Khalid’s (2020) review of strategies to improve the uptake of research evidence in different organisational contexts within low- and middle income settings highlighted the need for KE strategies that are appropriate across different sectors and organisations involved in response efforts [24]. Other researchers have also described barriers and facilitators affecting the success of KE. For example, Kayabu and Clarke (2013) found that those involved in disaster responses wanted to access and use evidence in decision making, but were hampered by lack of access, time and the required skills to interpret information [25]. In addition, and related to the previous point, crisis situations are recognised as requiring a range of different types of knowledge to ensure effective responses. These knowledges include the use of local and contextually relevant knowledge to a particular country or geography [25]. Further considerations include the complexities of accessing, sharing and using knowledge during emergency situations where decisions need to be made quickly [26].

Despite the critical need for effective knowledge exchange strategies to support crisis responses, as far as we are aware, no existing reviews have considered KE strategies that are inclusive of a broader range of knowledge than research evidence alone. In addition, previous reviews have not systematically mapped different models of KE across crisis types. To better understand KE processes within crises contexts, we conducted a scoping review with the aim of comparing different KE strategies across stages and types of crises. To conduct this comparison, we focused on the following questions: 1) What models of KE are utilised in crisis settings?; 2) What types of knowledge on the social determinants of health are exchanged in crisis settings?; 3) What are the processes, mechanisms and activities of KE in crisis settings?; 4) What are the barriers and facilitators to KE in crisis settings?; 5) What is the effectiveness of KE efforts in crisis settings?

## Methods

We conducted a scoping review broadly following the Arksey and O’Malley framework, which involves five key stages: 1) identifying the research questions (above); 2) identifying relevant studies; 3) selecting studies; 4) charting the data; and 5) collating, summarising and reporting results [27]. The PRISMA Scoping Reviews (PRISMA-ScR) Checklist is contained as a S1 Checklist.

### Identifying relevant studies

Relevant studies were identified through electronic searching of the Medline, EMBASE, Global Health, Scopus and Web of Science databases using terms and synonyms for crisis, public health/social determinants of health and knowledge exchange. The search was run in November 2020 with no date limits applied; the search was restricted to English-language publications. An example search strategy can be found in S1 File.

### Selecting studies

All identified records were imported into EndNote20 and duplicates were removed [28]. The remaining records were imported into Covidence for screening [29]. An initial 10% of titles and abstracts were reviewed by two reviewers to test the application of the inclusion criteria (below). The remaining abstracts were screened independently by one reviewer (EH or EM). Possibly relevant articles were retrieved for full text screening; 10% of full text articles were dual screened (by EH and EM) and the remainder were screened by one reviewer.

The following inclusion criteria were applied to each record (see Table 2): 1) article develops or describes KE, data access or evidence utilisation model, analyses process or evaluates effectiveness of KE; 2) the KE described is explicit rather than implicit; 3) the study is conducted within a crisis context; 4) the study topic is relevant to the social determinants of health and/or community health; 5) the article is a peer-reviewed publication or full-text conference paper; and 6) the paper is reported in the English language. Studies in any high, middle or low-income setting and utilising any study design were eligible for inclusion. Studies that focused exclusively on non-community based clinical settings (e.g. secondary and tertiary care) were excluded. Some research activities are focused on informing the public about risk and risky behaviours. Risk communication can take place in a range of contexts, including, for examples, infectious diseases/pandemics, environmental disasters, nuclear hazards and bioterrorism. Potentially, this includes a large amount of literature on health promotion, but only some of this literature meets the inclusion criteria for this review. Studies were eligible for inclusion where risk communication was reported alongside knowledge exchange and/or where knowledge exchange was undertaken to inform the development of a risk communication approach. In practice however, we recognise that the concepts of risk communication and knowledge exchange may be blurred and overlapping, given that both processes involve the flow of information to ‘individuals, groups and institutions’ whether to inform decisions at a personal, organisational or government level [30].

**Table 2:** Inclusion criteria.

Criteria	Description
KE data access or evidence utilisation model, KE processes or evaluation of KE	Article describes or develops a model of KE data access or evidence use, describes KE processes or evaluates the effectiveness of KE efforts, including broader political, social, economic and social determinants of evidence and knowledge use or non-use
Explicit KE	Explicit KE refers to studies in which the structures, resources or processes for KE are described; implicit KE refers to papers reporting on the exchange and/or use of knowledge within the context of decision making/planning or delivery of crisis management responses but without evidence of an explicit KE model in place or description of KE mechanisms
Crisis context	Any stage of a crisis, including mitigation, preparedness, response and including humanitarian crises, natural disasters, technical, terror or economic related crises or pandemics. Social movements such as Me Too and Black Lives Matter were considered as broader system changes and therefore not eligible for inclusion
Social determinants of heath or community health	Studies concerns upstream drivers and determinants of population health
Publication type	Peer-reviewed article or full conference paper
Language	English-language

We took a deliberately broad view of knowledge, including research evidence as well as scientific, epidemiological and surveillance data; formalised knowledge (e.g. guidelines or best practice); policy and professional contextual knowledge; and lay knowledge (e.g. lived expertise of communities of interest/place or the general public affected by crises).

### Charting the data

Guided by the review’s sub-questions, a form was developed and piloted to chart the data from each study. The form was piloted on 10% of studies independently by two reviewers (EH or EM) and the remainder of included studies were split between the two reviewers. Data were extracted on (i) the study aim; (ii) design; (iii) type of crisis; (iv) stage of crisis (Table 1); (v) model of KE [*‘evidence access’* referred to papers in which the KE model aimed to improve accessibility of research evidence (e.g. a knowledge hub) or access to data and *‘Active KE’* referred to papers reporting mechanisms planned, or in place, to support active exchange of knowledge between stakeholders as part of crisis responses]; (vi) type and description of knowledge exchanged (research evidence/data; practitioner/policy; and/or lay/public); (vii) if the KE takes place during crisis, happens retrospectively or it is a conceptual paper describing KE for a crisis context; (viii) definitions, models and frameworks of KE; (ix) purpose or rationale for KE; (x) mechanisms and activities in place to support KE; (xi) barriers and facilitators to KE; (xii) changes brought about by KE processes and activities; (xiii) KE recommendations and (xiv) researchers’ notes. Although our synthesis and findings were guided by the theories of KE outlined in the introduction, we did not impose a pre-defined KE framework when extracting and synthesising data. Rather, we sought to surface the theories of how KE has been understood in different crisis contexts.

### Collating, summarising and reporting results

The charted data were then analysed and synthesised, guided by the review’s overarching comparative aim and sub-questions. Specifically, we developed a descriptive understanding of the study’s characteristics and then identified the types of KE, barriers and facilitators to KE and KE recommendations, paying specific attention to how these vary across different types and stages of crisis. The results are presented in both tabular and narrative form. Matrices 1 and 2 summarise the number of studies by stage of crisis and model of KE (Matrix 1) and by type of crisis and model of KE (Matrix 2). Table 3 provides an overview of each study’s characteristics, including setting and description of knowledge exchanged. Table 4 distils the KE mechanisms and activities, types of knowledge and underlying KE theories across crisis stages and types of crises.

**Matrix 1:** Stage of crisis and model of KE (number of studies).

	Active KE	Evidence Access	Combined Approach of KE
**Mitigation**	9	10	7
**Preparedness**	14	17	10
**Response**	21	32	17
**Recovery**	7	9	6

**Matrix 2:** Type of crisis and model of KE (number of studies).

	Active KE	Evidence Access	Combined Approach of KE
**Pandemic**	8	23	5
**Environmental**	14	2	6
**Terrorism**	0	1	1
**Technical**	0	1	1
**Humanitarian**	0	1	1
**Various**	8	8	6

**Table 3:** Overview of study characteristics by crisis types.

Study	Country / ies	Methods	Type of crisis	Stage of crisis	Real life or conceptual crisis	Model of KE	Types of knowledge exchanged
**Pandemics**							
**Adini et al. (2019) *Earlier detection of public health risks—Health policy lessons for better compliance with the International Health Regulations (IHR 2005)*: *Insights from low-*, *mid- and high-income countries* [31]**	Israel	Workshop	Pandemic (SARS; H1N1; Ebola; Zika)	Preparedness	Conceptual	Active KE	Research evidence / data Practitioner / policy knowledge
**Akselrod et al. (2012) *Creating a process for incorporating epidemiological modelling into outbreak management decisions* [32]**	United States	Project description and framework development	Pandemic (infectious diseases)	Response	Conceptual	Active KE	Research evidence / data Practitioner / policy knowledge
**Ammirato et al. (2020) *Knowledge management in pandemics. A critical literature review [33]***	Global	Literature review	Pandemic (various)	All stages implied	Conceptual	Evidence Access	Research evidence / data
**Baker and Forsyth (2007) *The new international health regulations*: *A revolutionary change in global health security* [34]**	New Zealand (and global)	Descriptive account of IHR revisions	Pandemics (infectious disease)	Preparedness; Response (both implicit)	Conceptual	Evidence Access	Research evidence / data
**Bdeir et al. (2017) *Informal networks in disaster medicine* [35]**	Australia	Social network analysis (qualitative and quantitative surveys; interviews)	Pandemic (infectious diseases)	Preparedness; Response	Real life crisis	Active KE	Practitioner / policy knowledge
**Boyd et al. (2010) *The use of public health grid technology in the United States Centers for Disease Control and Prevention H1N1 pandemic response* [36]**	United States	Descriptive case study	Pandemic (H1N1)	Response	Real life crisis	Evidence Access	Research evidence / data Practitioner / policy knowledge
**Briand et al. (2011) *Challenges of global surveillance during an influenza pandemic* [37]**	Global	Workshop	Pandemic (Influenza/H1N1)	Preparedness; Response	Real life crisis	Combined approach	Research evidence / data Practitioner / policy knowledge
**Brookes et al. (2015) *Preparedness for emerging infectious diseases*: *pathways from anticipation to action* [38]**	Global	Desk based review	Pandemic (infectious diseases)	Preparedness	Conceptual	Combined approach	Research evidence / data Practitioner / policy knowledge
**Caceres et al. (2017) *The World Organisation for Animal Health and the World Health Organization*: *intergovernmental disease information and reporting systems and their role in early warning* [39]**	WHO and OIE Member States	Descriptive case studies	Pandemics (infectious diseases)	Preparedness	Conceptual	Evidence Access	Research evidence / data Practitioner / policy knowledge
**Carrion ProMED-mail: 22 years of digital surveillance of emerging infectious diseases [40]**	Global	Descriptive account of service	Pandemic (SARS, MERS; Ebola; Zika)	Preparedness	Real life crisis	Evidence Access	Research evidence / data Practitioner / policy knowledge Community / public knowledge
**Colf et al. (2016) *A role for science in responding to health crises* [41]**	United States	Descriptive case study	Pandemic (Ebola)	Response	Real life crisis	Evidence Access	Research evidence / data
**Dearinger et al. (2011) *Communication efforts among local health departments and health care professionals during the 2009 H1N1 outbreak* [42]**	United States	Cross-sectional survey	Pandemic (H1N1)	Response	Real life crisis	Evidence Access	Research evidence / data Practitioner / policy knowledge
**Driedger et al. (2014) *Developing model-based public health policy through knowledge translation*: *the need for a ’Communities of Practice’* [43]**	Canada	Interviews; workshops	Pandemic (H1N1)	Preparedness, Response	Real life crisis	Active KE	Research evidence / data Practitioner / policy knowledge
**El-Jardali et al. (2020) *Amplifying the role of knowledge translation platforms in the COVID-19 pandemic response* [44]**	Lebanon	Descriptive account	Pandemic (COVID-19)	Response	Real life crisis	Evidence Access	Research evidence / data Practitioner / policy knowledge
**Fangman et al. (2015) *Routine dissemination of summary syndromic surveillance data leads to greater usage at local health departments in North Carolina* [45]**	United States	Interviews; survey	Pandemic (H1N1)	Response	Real life crisis	Combined approach	Research evidence / data
**Kabad et al. (2020) *The experience with volunteer and collaborative work in mental health and psychosocial care during the COVID-19 pandemic* [46]**	Brazil	Descriptive account	Pandemic (COVID-19)	Response	Real life crisis	Evidence Access	Research evidence / data Practitioner / policy knowledge
**Khan et al. (2010) The next public health revolution: public health information fusion and social networks [47]**	United States	Descriptive account	Pandemic (public health threats e.g. SARS, H1N1)	Mitigation, Preparedness, Response	Conceptual	Evidence Access	Research evidence / data Practitioner / policy knowledge
**Liverani et al. (2018) *Sharing public health data and information across borders*: *lessons from Southeast Asia* [48]**	Cambodia and Vietnam	Key informant interviews	Pandemic (infectious diseases)	Preparedness, Response	Both	Active KE	Research evidence / data Practitioner / policy knowledge
**Maddox and Grapsa (2020) *Developing credible knowledge during the COVID-19 pandemic*: *experiences with JACC*: *case reports and the ACC COVID-19 Hub* [49]**	United States	Descriptive account	Pandemic (COVID-19)	Response	Real life crisis	Evidence Access	Research evidence / data Practitioner / policy knowledge
**Majumder and Mandl (2020) *Early in the epidemic*: *impact of preprints on global discourse about COVID-19 transmissibility* [50]**	Global	Literature review including searches of pre-prints	Pandemic (COVID-19)	Response	Real life crisis	Evidence Access	Research evidence / data
**Modjarrad et al. (2016) *Developing global norms for sharing data and results during public health emergencies* [51]**	Global	Consultation workshop	Pandemic (public health emergencies)	Response	Conceptual	Evidence Access	Research evidence / data
**Moore et al. (2020) *Ideas for how informaticians can get involved with COVID-19 research* [52]**	Global	Descriptive case study	Pandemic (COVID-19)	Response	Both	Evidence Access	Research evidence / data Practitioner / policy knowledge Community / public knowledge
**Nabyonga-Orem et al. (2016) *Assessing policy dialogues and the role of context*: *Liberian case study before and during the Ebola outbreak* [53]**	Liberia	Semi structured interviews	Pandemic (Ebola)	Response	Real life crisis	Active KE	Research evidence / data Practitioner / policy knowledge Community / public knowledge
**Omange et al. (2017) *Meeting report*: *Unesco-Merck Africa research summit 2015- accelerating access and sustaining innovation ‘From Africa for Africa’* [54]**	Multiple African countries	Descriptive account	Pandemic (Ebola and others)	Preparedness, Response	Both	Combined approach	Research evidence / data
**Powell et al. (2018) *The role of knowledge in system risk identification and assessment*: *the 2014 Ebola outbreak* [55]**	West African countries	Qualitative system dynamics modelling	Pandemic (Ebola)	Response	Real life crisis	Active KE	Research evidence / data Practitioner / policy knowledge Community / public knowledge
**Quinn et al. (2018) *Variations in healthcare provider use of public health and other information sources by provider type and practice setting during New York City’s response to the emerging threat of Zika Virus Disease*, *2016* [56]**	United States	Cross-sectional survey	Pandemic (Zika)	Response	Real life crisis	Evidence Access	Research evidence / data Practitioner / policy knowledge
**Sakusic et al. (2020) *Rapid*, *multimodal*, *critical care knowledge-sharing platform for COVID-19 pandemics* [57]**	Southeastern Europe	Online survey	Pandemic (COVID-19)	Response	Real life crisis	Combined approach	Research evidence / data Practitioner / policy knowledge
**Sen et al. (2020) *Reflections on social work 2020 under Covid-19 online magazine* [58]**	Multiple (HMICs)	Descriptive account	Pandemic (COVID-19)	Response	Real life crisis	Evidence Access	Research evidence / data Practitioner / policy knowledge Community / public knowledge
**Shapiro et al. (2010) *Health information exchange*, *biosurveillance efforts*, *and emergency department crowding during the spring 2009 H1N1 outbreak in New York City* [59]**	United States	Analysis of routine data; descriptive account	Pandemic (H1N1)	Preparedness, Response	Real life crisis	Evidence Access	Research evidence / data
**Smith et al. (2017) *Knowledge sharing in global health research—the impact*, *uptake and cost of open access to scholarly literature* [60]**	Global	Literature search; review of open access research	Pandemic (general)	Mitigation, Response	Conceptual	Evidence Access	Research evidence / data
**Spagnolo et al. (2020) *Reflecting on knowledge translation strategies from global health research projects in Tunisia and the Republic of Cote d’Ivoire* [61]**	Cote d’Ivoire	In-depth interviews	Pandemic (Ebola)	Mitigation	Real life crisis	Active KE	Research evidence / data Practitioner / policy knowledge Community / public knowledge
**Taylor and Stephenson (2009) *Influenza A (H1N1) virus (swine influenza)*: *a webliography* [62]**	United States (and global)	Descriptive account	Pandemic (H1N1)	Response	Real life crisis	Evidence Access	Research evidence / data Practitioner / policy knowledge
**Utunen (2020) *Serving health emergency responders through online learning—findings from OpenWho’s global user metrics* [63]**	Global	Analysis of platform data	Pandemic (general)	Response, Recovery	Both	Evidence Access	Research evidence / data Practitioner / policy knowledge
**Utunen (2018) *Knowledge transfer for Ebola outbreak—production and use of OpenWHO*.*org online learning resources* [64]**	Democratic Republic of the Congo	Descriptive account	Pandemic (Ebola)	Response, Recovery	Real life crisis	Evidence Access	Practitioner / policy knowledge
**Valaitis et al. (2005) *A Severe Acute Respiratory Syndrome extranet*: *supporting local communication and information dissemination* [65]**	Canada	Survey	Pandemic (SARS)	Preparedness, Response	Real life crisis	Evidence Access	Research evidence / data Practitioner / policy knowledge
**Zhang et al. (2017) *Knowledge management framework for emerging infectious diseases preparedness and response*: *design and development of public health document ontology* [66]**	Global	Descriptive account	Pandemic (general)	Preparedness, Response	Conceptual	Evidence Access	Research evidence / data Practitioner / policy knowledge
**Environmental crises**							
**Alexander et al. (2014) *Translating the complexities of flood risk science using KEEPER—a knowledge exchange exploratory tool for professionals in emergency response [67]***	United Kingdom	Development of tool (interviews); demonstration of tool; evaluation of tool (interviews; questionnaires)	Environmental (flooding)	All stages	Conceptual	Active KE	Research evidence / data Practitioner / policy knowledge
**Anderson et al. (2007) *Reducing landslide risk in areas of unplanned housing in the Caribbean—a government-community partnership model* [68]**	Saint Lucia	Programme description	Environmental (landslips)	Mitigation; Response	Real life crisis	Active KE	Research evidence / data Practitioner / policy knowledge Community / public knowledge
**Ballé-Béganton et al. (2012) *Building an integrated model for freshwater allocation with local managers in a coastal area* [69]**	France	Descriptive account of building an integrated model using a ecosystem services approach	Environmental (low river flows)	Response	Conceptual	Active KE	Research evidence / data Practitioner / policy knowledge
**Blake et al. (2019) *The role of data and information exchanges in transport system disaster recovery*: *a New Zealand case study* [70]**	New Zealand	Interviews	Environmental (earthquake)	Response; Recovery; Mitigation	Real life crisis	Combined approach	Research evidence / data Practitioner / policy knowledge
**Crane et al. (2017) *Use of information and communication technologies in the formal and informal health system responses to the 2015 Nepal earthquakes* [71]**	Nepal	In-depth semi-structured interviews; focus groups	Environmental (earthquake)	Response	Real life crisis	Active KE	Research evidence / data Community / public knowledge
**El Amiri et al. (2020) *Community of practice*: *an effective mechanism to strengthen capacity in climate change and health* [72]**	Canada	Descriptive case study	Environmental (climate change)	Mitigation, Response	Real life crisis	Combined approach	Research evidence / data Practitioner / policy knowledge Community / public knowledge
**Hayles (2010) *An examination of decision making in post disaster housing reconstruction* [73]**	Global	Literature review; survey	Environmental (various)	Recovery	Both	Active KE	Practitioner / policy knowledge Community / public knowledge
**Hendriks and Opdyke (2020) *Knowledge adoption in post-disaster housing self-recovery* [74]**	Philippines	Semi-structured interviews; focus groups	Environmental (typhoon)	Recovery	Real life crisis	Active KE	Practitioner / policy knowledge Community / public knowledge
**Hidayat and Afif (2017) *Knowledge transfer to builders in post-disaster housing reconstruction in West-Sumatra of Indonesia* [75]**	Indonesia	Semi-structured interviews	Environmental (earthquakes)	Recovery, Mitigation	Real life crisis	Evidence Access	Research evidence / data
**Huang et al. (2010) *Web 2*.*0 and internet social networking*: *a new tool for disaster management*?*—Lessons from Taiwan* [76]**	Taiwan	Descriptive country case study	Environmental (typhoon)	Response	Real life crisis	Active KE	Community / public knowledge
**Ingirige et al. (2008) *Exploring good practice knowledge transfer related to post-tsunami housing (re-)construction in Sri Lanka* [77]**	Sri Lanka	Documentary review; survey	Environmental (tsunami)	Recovery	Real life crisis	Active KE	Practitioner / policy knowledge Community / public knowledge
**Lemos et al. (2020) *Building on adaptive capacity to extreme events in Brazil*: *water reform*, *participation*, *and climate information across four river basins* [78]**	Brazil	Documentary review; interviews; participatory observation	Environmental (climate events)	Mitigation, Preparedness, Response	Real life crisis	Combined approach	Research evidence / data Practitioner / policy knowledge Community / public knowledge
**Nakano et al. (2020) *Long-term evaluation of proactive attitudes toward disaster education in Nepal* [79]**	Nepal	Literature review; longitudinal participant observation	Environmental (earthquake)	Preparedness, Mitigation, Response	Real life crisis	Active KE	Community / public knowledge
**Nakatani et al. (2006) *Three examples of disaster damage mitigation from the viewpoint of information* [80]**	Japan	Descriptive case study	Environmental (earthquakes; typhoons; floods; landslides; heavy snows; volcanic explosions)	Mitigation, Response	Conceptual	Combined approach	Research evidence / data Practitioner / policy knowledge Community / public knowledge
**Oktari et al. (2015) *A conceptual model of a school-community collaborative network in enhancing coastal community resilience in Banda Aceh*, *Indonesia* [81]**	Indonesia	Literature review; focus groups; questionnaire	Environmental (mostly tsunami)	Mitigation, Preparedness, Response	Both	Active KE	Research evidence / data Practitioner / policy knowledge Community / public knowledge
**Powell (2011) *Post-disaster reconstruction*: *a current analysis of Gujarat’s response after the 2001 earthquake* [82]**	India	Semi-structured interviews; visual surveys; questionnaires	Environmental (earthquake)	Mitigation, Response, Recovery	Real life crisis	Active KE	Practitioner / policy knowledge Community / public knowledge
**Reyers et al. (2015) *Navigating complexity through knowledge coproduction*: *mainstreaming ecosystem services into disaster risk reduction* [83]**	South Africa	Interviews; meetings; literature reviews; policy analysis; field trips	Environmental (flood; wildfire; drought; storm waves)	Mitigation	Both	Active KE	Research evidence / data Practitioner / policy knowledge Community / public knowledge
**Shrestha et al. (2013) *The impact of retrofitting work on awareness raising and knowledge transfer in Aceh Province*, *Indonesia* [84]**	Indonesia	Interviews; survey	Environmental (earthquake; tsunami)	Mitigation	Real life crisis	Active KE	Practitioner / policy knowledge Community / public knowledge
**Simich et al. (2008) *Post-disaster mental distress relief*: *Health promotion and knowledge exchange in partnership with a refugee diaspora community* [85]**	Canada	Workshop	Environmental (tsunami)	Preparedness, Response, Recovery	Real life crisis	Active KE	Research evidence / data Practitioner / policy knowledge Community / public knowledge
**Thanurjan and Senevirante (2009) *The role of knowledge management in post-disaster housing reconstruction* [86]**	Sri Lanka	Literature review; semi-structured interviews	Environmental (tsunami; draught; rock falls; tropical storms; fires; landslides; high wind; floods)	Response, Recovery	Both	Combined approach	Practitioner / policy knowledge
**Wistow et al. (2017) *Implementing extreme weather event advice and guidance in English public health systems* [87]**	United Kingdom (England)	Focus groups	Environmental (extreme weather events)	Preparedness, Mitigation	Conceptual	Evidence Access	Research evidence / data Practitioner / policy knowledge
**Yahya et al. (2015) *Towards an essential knowledge transfer process model in the flood management domain* [88]**	Malaysia	Literature review; development of conceptual model	Environmental (flood)	Preparedness, Response	Conceptual	Combined approach	Practitioner / policy knowledge
**Humanitarian crises**							
**Bolisani and Damiani (2010) *Knowledge management in complex environments*: *the UN peacekeeping* [26]**	Countries with UN peacekeeping presence	Participant observation	Humanitarian	Response; Recovery	Real life	Combined approach	Research evidence / data Practitioner / policy knowledge
**Codjia et al. (2018) *Enhancing infant and young child feeding in emergency preparedness and response in East Africa*: *capacity mapping in Kenya*, *Somalia and South Sudan* [89]**	East African countries (Kenya, Somalia and South Sudan)	Capacity mapping exercise (desk review; interviews; stakeholder consultation)	Humanitarian	Preparedness, Response	Real life crisis	Evidence Access	Research evidence / data Practitioner / policy knowledge
**Technical crises**							
**Ionita et al. (2019) *Knowledge-based education and awareness about the radiological and nuclear hazards* [90]**	Countries with nuclear power plants	Descriptive account	Technical (radiological; nuclear)	Mitigation, Response	Conceptual	Evidence Access	Research evidence / data
**Raskob et al. (2015) *PREPARE*: *innovative integrated tools and platforms for radiological emergency preparedness and post-accident response in Europe* [91]**	Europe	Descriptive account	Technical (nuclear; radiological)	Preparedness	Conceptual	Combined approach	Research evidence / data Practitioner / policy knowledge
**Terror related crises**							
**Anosike (2018) *Entrepreneurship education knowledge transfer in a conflict Sub-Saharan African context* [92]**	Nigeria	In-depth interviews	Terror related conflict	Recovery; Mitigation	Real life crisis	Combined approach	Research evidence / data Community / public knowledge
**Ferguson et al. (2003) *Bioterrorism web site resources for infectious disease clinicians and epidemiologists* [93]**	United States	Survey; review of academic databases and online discussion forums	Bioterrorism	Preparedness, Response	Conceptual	Evidence Access	Research evidence / data Practitioner / policy knowledge
**Various crises**							
**Allen (2014) *A resource for those preparing for and responding to natural disasters*, *humanitarian crises*, *and major healthcare emergencies* [94]**	Global	Description of the information platform’s background and development	Various	All stages	Real life crisis	Evidence Access	Research evidence / data
**Benis et al. (2018) *Risk and disaster management*: *from planning and expertise to smart*, *intelligent*, *and adaptive systems* [95]**	Israel	Descriptive case study	Various (Pandemic; environmental; technical; humanitarian)	All stages	Conceptual	Evidence Access	Research evidence / data
**Biddinger et al. (2010) *Public health emergency preparedness exercises*: *lessons learned* [96]**	United States	Evaluation (content analysis of plans; pre/post and post surveys)	Various (general public health threats)	Preparedness; Response	Real life crisis	Active KE	Research evidence / data Practitioner / policy knowledge
**Borell and Eriksson (2008) *Improving emergency response capability*: *an approach for strengthening learning from emergency response evaluations* [97]**	Sweden	Documentary review; interviews	Various (Humanitarian, environmental)	Preparedness; Response	Real life crisis	Active KE	Research evidence / data Practitioner / policy knowledge
**De Brun (2017) *What is the evidence around knowledge and library service provision and knowledge management to support global health*, *and disaster and emergency preparedness*? [98]**	Global	Literature review	Various (climate change; natural disasters)	Preparedness. Response	Conceptual	Evidence Access	Research evidence / data Practitioner / policy knowledge
**Canós et al. (2010) *Using spatial hypertext to visualize composite knowledge in emergency responses* [99]**	Spain	Descriptive account of framework’s development	Various	Response	Conceptual	Active KE	Research evidence / data Practitioner / policy knowledge Community / public knowledge
**Carroll and Madoff (2017) *Examples of applied public health through the work of the Epidemic Intelligence Service officers at CDC’s National Center for Environmental Health*: *2006–2015* [100]**	United States	Documentary review; qualitative interviews	Various (Natural disasters; toxic chemicals; extreme temperature)	Preparedness, Response	Real life crisis	Evidence Access	Research evidence / data
**Cheng and Wu (2006) *Data exchange platform for bridge disaster prevention using intelligent agent* [101]**	Taiwan	Descriptive account of platform’s development	Various (environmental; technical)	All stages	Conceptual	Evidence Access	Research evidence / data
**Généreux et al. (2019) *From science to policy and practice*: *a critical assessment of knowledge management before*, *during*, *and after environmental public health disasters* [102]**	Canada	In-depth interviews; observations; documentary review	Various environmental and technical (wildfires and chemical spills)	All stages	Both	Combined approach	Research evidence / data Practitioner / policy knowledge Community / public knowledge
**Khalid et al. (2020) *Supporting the use of research evidence in decision-making in crisis zones in low- and middle-income countries*: *a critical interpretive synthesis* [24]**	Multiple (LMICs)	Systematic review	Various (humanitarian; environmental)	All stages	Real life crisis	Combined approach	Research evidence / data Practitioner / policy knowledge
**Lenart et al.(2012) *Integrating public health and medical intelligence gathering into homeland security fusion centres* [103]**	United States	Descriptive case study	Various (natural and terrorist-related threats)	Response	Both	Active KE	Practitioner / policy knowledge
**Lu et al. (2013) *Learning mechanisms for humanitarian logistics* [104]**	Multiple (LMICs)	Literature review; development of conceptual framework	Various (environmental; humanitarian)	All stages	Conceptual	Evidence Access	Practitioner / policy knowledge Community / public knowledge
**Mellon (2015) *Evaluating Evidence Aid as a complex*, *multicomponent knowledge translation intervention* [105]**	Global	Descriptive case study; presentation of conceptual framework	Various (natural or man-made; complex emergency / conflict)	Preparedness, Response	Conceptual	Combined approach	Research evidence / data Practitioner / policy knowledge
**Modigell and Khara (2019) *Global technical assistance mechanism for nutrition (GTAM)*: *the story so far* [106]**	Global	Case study drawing on existing studies, reports; interviews	Various	Response	Conceptual	Combined approach	Research evidence / data Practitioner / policy knowledge
**Pathirage et al. (2012) *Managing disaster knowledge*: *identification of knowledge factors and challenges* [107]**	Global	Literature review; semi-structured interviews	Various	All stages	Conceptual	Active KE	Practitioner / policy knowledge
**Qari et al. (2018) *Overview of the translation*, *dissemination*, *and implementation of public health preparedness and response research and training initiative* [108]**	United States	Review of projects	Various (pandemic; environmental; technical)	Preparedness, Response	Conceptual	Combined approach	Research evidence / data Practitioner / policy knowledge
**Revere and Fuller (2008) *Building a customizable knowledge management environment to support public health practice*: *design strategies* [109]**	United States	Literature review	Various (pandemic; bioterrorism; environmental disasters)	Response	Conceptual	Evidence Access	Research evidence / data Practitioner / policy knowledge
**Savoia et al. (2012) *Use of after action reports (AARs) to promote organizational and systems learning in emergency preparedness* [110]**	United States	Structured review	Various (pandemic H1N1; environmental hurricanes)	Response, Preparedness	Real life crisis	Active KE	Practitioner / policy knowledge
**Stevens et al. (2017) *Knowledge exchange for resource management and international trust (KERMIT)) Aleppo case-study example* [111]**	Syria	Descriptive case study	Various intersecting (humanitarian; environmental; technical)	Response	Real life crisis	Active KE	Research evidence / data Practitioner / policy knowledge Community / public knowledge
**Stoddart et al. (2015) *Developing a knowledge strategy for medical humanitarian crises*: *a case study of Médecins Sans Frontiéres (MSF)*, *Switzerland* [112]**	Multiple (LMICs)	Interviews	Various (including Ebola)	Response	Both	Combined approach	Research evidence / data Practitioner / policy knowledge
**Turner et al. (2011) *Supporting evidence-based health care in crises*: *what information do humanitarian organizations need*? [113]**	Global	Semi-structured interviews	Various (environmental; humanitarian)	Response, Recovery	Conceptual	Evidence Access	Research evidence / data
**Wells et al. (2013) *Applying community engagement to disaster planning*: *Developing the vision and design for the Los Angeles county community disaster resilience initiative* [114]**	United States	Semi structured interviews; description of evaluation plan	Various (pandemic; environmental; technical)	Preparedness	Conceptual	Active KE	Research evidence / data Practitioner / policy knowledge Community / public knowledge

Abbreviations: LMICs: Low- and middle-income countries; HMICs: High- and middle-income countries.

**Table 4:** Knowledge exchange across crisis types and stages.

	Examples of KE activities	Types of knowledge	Underlying theories and principles
**Environmental**	*Preparedness; mitigation*:• Multi-component KE in community settings (e.g., between researchers, schools, public, government officials) to reduce risk of environmental hazards • Systems approaches that facilitate integration of knowledge(s) on policy issues (e.g., weather related events) • Communities of practice (e.g., to tackle climate change)*Response*: • Informal and social networks to exchange informal knowledge during immediate crisis situations • Intra and inter-organisational structures (e.g. crisis response forums)*Recovery*: • NGOs or donor programmes to support safer construction practices	Formalised knowledge (e.g. technical guidance on housing construction); multi-sectorial and disciplinary perspectives including organisational and policy/professional knowledge; local and contextual knowledge of volunteers and communities; scientific data and research evidence.	Co-creation of knowledge between multiple stakeholders including the public. Participatory and collaborative approaches to KE
**Humanitarian**	*Preparedness; response* • Organisational KE strategies including employment of KE professionals; knowledge management systems; translation of best practices and turning learning from crises into formalised knowledge.	Research evidence; formalised knowledge (best practice, guidance, manuals) experiential knowledge; tacit knowledge of crisis response staff	Organisational knowledge management; knowledge exchange between researchers and aid professionals
**Pandemics**	*Preparedness; mitigation* • Interactive systems modelling • Workshops *Response* • Public or member-only repositories (e.g. websites, hubs, portals) • Free access to journals/preprints • Rapid response teams/services • Communication (e.g email lists and bulletins) to share latest outbreak information and evidence • Internet-based resources • Cross-border procedures and networks *Recovery* • Online learning materials and resources for professionals	Research evidence, surveillance/ epidemiological data and/or formalised knowledge (e.g. public health guidelines); policy and professional contextual knowledge. Limited reference to lay knowledge with exception of Ebola outbreaks.	Emphasis on translation and exchange of research evidence and data for policy decision making and action. Use of social network and systems theories referenced.
**Technical**	*Preparedness; mitigation*, *response* • Web based resources and information	Research evidence and scientific data; formalised knowledge (e.g., guidelines); knowledge of NGOs, governmental organisations and scientists	Not explicitly stated
**Terrorism**	*All stages* • Web based resources and information • University programmes and outreach placements to support economic recovery	Formalised knowledge (e.g. case studies, fact sheets); published research findings; pathological, infection control / clinical / epidemiological data; academic expertise and experiential knowledge of communities	Not stated explicitly

## Results

**Fig 1:**
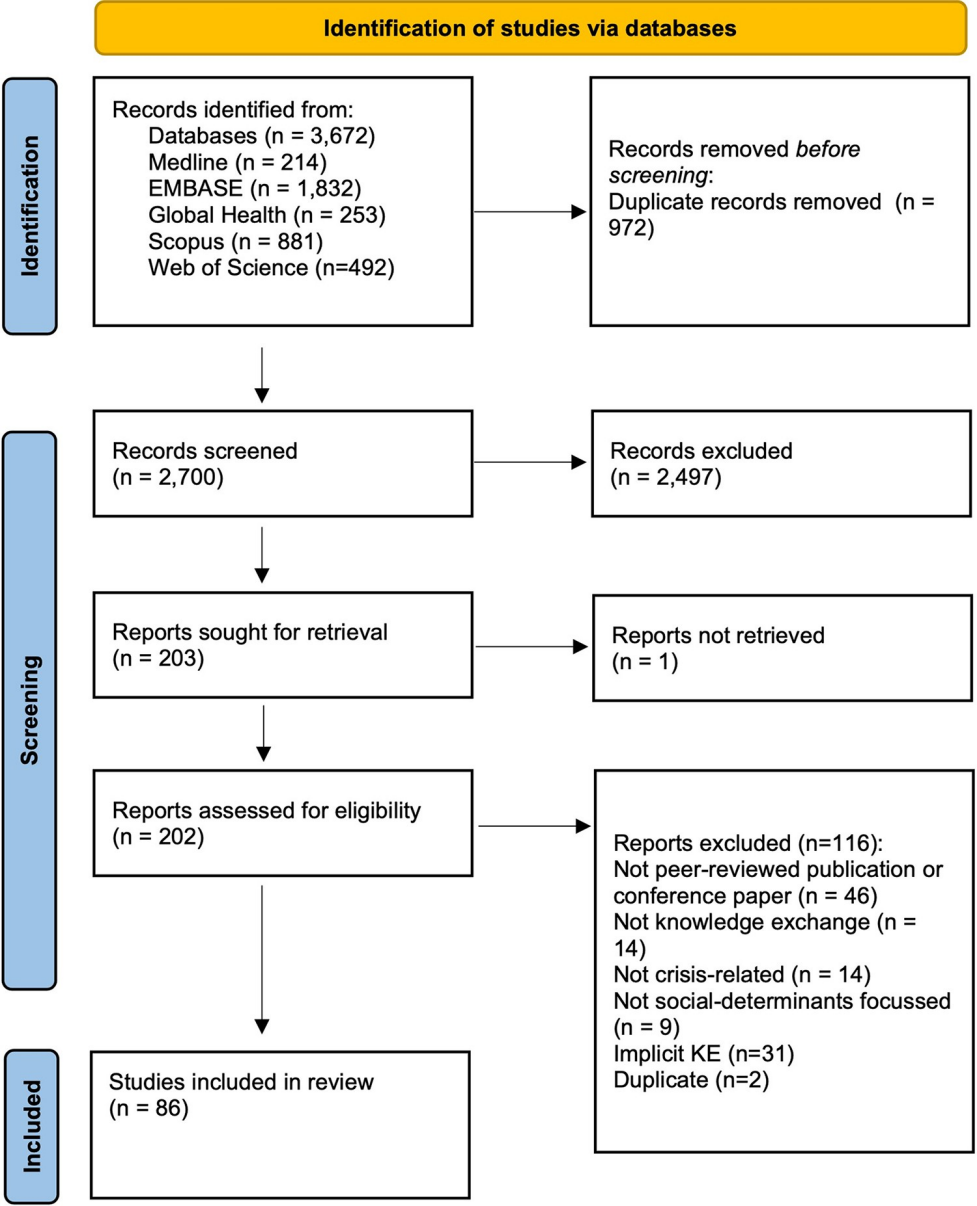
PRISMA 2020 flow diagram.

A total of 86 studies were identified for inclusion in the review and the flow of studies through the study selection process is depicted in Fig 1 [115]. For readability, references have been removed from the following section (overview of study characteristics), however, characteristics for each study are detailed in Table 3.

### Overview of study characteristics

#### Settings for KE, geographical and crisis types/stages

Thirty-six studies were set in high income country contexts including Australia, Canada, France, Israel, Japan, New Zealand, Spain, Sweden, Taiwan, the UK and the USA. Seventeen studies were conducted in middle-income settings, including Brazil, Côte d’Ivoire, India, Indonesia, Lebanon, Malaysia, Nigeria, Nepal, Philippines, Sri Lanka, St Lucia and South Africa. Three studies were conducted in low-income settings in Democratic Republic of the Congo Liberia and Syria. Thirty studies reported on KE in multiple country settings, which included papers with a global focus or a focus on low-and middle income countries, high- and middle-income countries, African countries, European countries, Southeast Asian countries, countries with a UN peacekeeping presence or countries with nuclear power plants.

KE was most frequently designed for, or undertaken in the context of, pandemic-related crises (n = 36) (e.g. Covid-19, Ebola, H1N1, various infectious disease outbreaks). Of these, 7 focused specifically on the Covid-19 pandemic. A large number of studies (n = 22) concerned KE conducted during environmental disasters (e.g. earthquakes, tsunamis, typhoons, flooding, landslides). A smaller number of studies described KE within the context of humanitarian conflicts (n = 2), technical disasters (e.g. nuclear and radiological disasters) (n = 2) and terror-related emergencies (n = 2), including bio-terrorism and ongoing terror related conflict. Twenty-two studies focused on more than one crisis type (e.g. pandemic, environmental, technical and humanitarian).

Approximately half (n = 39) studies considered KE at one crisis stage. The majority focussed on response with some focusing on preparedness, recovery or mitigation. The remaining studies (n = 47) explored KE at two or more crisis stages. In studies involving more than one crisis stage, KE was most frequently considered in context of the crisis response (n = 43) followed by preparedness (n = 32), mitigation (n = 23) and recovery stages (n = 19).

Forty-five studies reported on KE taking place in the context of a ‘real life’ crisis or retrospectively analysed a specific crisis, with an emphasis on reflecting on lessons learnt from these experiences. Thirty studies reported on the development of KE resources that had not been applied in real life settings (termed ‘conceptual papers’). Eleven studies included both conceptual KE and KE that was grounded within the context of a specific crisis or was informed by participants’ experiences of previous crises.

#### Study methods

The included studies drew on a range of different methods to describe and analyse KE processes and activities relevant to crisis settings. Study methods included surveys (n = 14) desk-based reviews and analysis of literature, documents or data (n = 27), qualitative methods including interviews, focus groups, stakeholder workshops and participant observation (n = 35), social network analysis (n = 1) and qualitative system dynamics modelling (n = 1). Thirty studies were descriptive case studies of KE programmes and projects in crisis settings.

### KE mechanisms and activities

#### KE overview

Thirty studies described models of active KE, whereas 36 focused on improving the accessibility of evidence or data. Twenty studies described and analysed models of KE that incorporated elements of both of these approaches.

The different types and sources of knowledge varied across studies, with many studies describing the exchange of multiple types of knowledge. The majority of studies (n = 70) described the exchange of research evidence and data [24,26,31–34,36–63,65–70,72,75,78,80,81,83,85,87,89–102,105,106,108,109,111–114]. The majority of studies (n = 67) also described the exchange and use of practitioner/policy knowledge or formalised knowledge such as guidance for practitioners or knowledge from organisational settings [24,26,31,32,35–40,42–44,46–49,52,53,55–58,61–74,77,78,80–89,91,93,96–99,102–112,114]. A third of studies (n = 27) involved the exchange and use of lay or public knowledge, including, for example, local beliefs about diseases and knowledge of local building construction processes [40,52,53,55,58,61,68,71–74,76–85,92,99,102,104,111,114].

Next, we report on the type and nature of KE by different crisis type providing a narrative account of this. Table 4 summarises examples of different KE activities, types of knowledge exchanged and underlying theories of KE across crises types and stages. Additionally, Matrix 1 tabulates the number of studies related to different models of KE by stage of crisis and Matrix 2 tabulates the model of KE by type of crisis.

### Pandemic crises

Evidence access approaches (n = 21) were largely situated during the response stage of a pandemic, aiming to support policy makers and professionals to access knowledge to inform decision making and their practice. Just under half of these studies (n = 11) reported the model of KE was, or could be, used during the preparedness [33,34,39,40,47,59,65,66]or mitigation stages [33,47,60]of a crisis. Few studies explicitly described a theoretical framework underpinning their evidence access model. Where mentioned, this included reference to knowledge management [33,66] or knowledge transfer models (e.g. dissemination of evidence) to inform policy decision making [44].

Evidence access approaches included public or member-only repositories (e.g. websites, hubs, portals) [49,52,62], the use of rapid evidence response teams/services [46,72]; free/open access to journals [50,58,60] and communication mechanisms such as email lists and bulletins [40,42,49,56]. Learning resources (e.g. online platforms) aimed to equip health workers to prepare for, and respond to, public health emergencies [63,64]. Within organisational settings, internal or member-only platforms facilitated access to knowledge available within and across organisations [36,47,59,65,66]. One study reported on KE activities to support pandemic responses at a national government level [41]. Three studies reported global level activities including norms, procedures and systems to facilitate access to surveillance data and evidence across borders [34,39,51].

Eight studies reported the use of active KE and concerned the response stage of a pandemic [31,32,35,43,48,53,55,61] involving mainly exchange between researchers and policy/practitioners [31,32,35,43,48]. Exchange involving lay knowledge was reported in three of these studies [53,55,61]. Where theoretical frameworks were referenced, this included knowledge translation aiming to address the knowledge to practice gap [43,48] and knowledge exchange [53,61], as well as the use of social network [35] and systems theory [55].

KE models involving researcher and practice/professional exchange included the use of interactive modelling and workshops bringing together research findings and data on disease and interventions with professional knowledge to inform decision making [31,32,43]. They also included the use of cross border networks to share data, information, and expertise [48]. An observational study of organisational KE reported on the role of informal networks and flows of knowledge between professionals during the H1N1 pandemic [35]. Three studies referred to KE approaches (e.g. participatory consensus workshops), which were inclusive of lived experiences of communities; each of these studies related to the Ebola crisis [53,55,61].

Five studies reported a combined KE approach at global and organisational levels; four focused on the response stage [37,45,54,57] with three studies also considering preparedness planning [37,38,54]. Types of knowledge within combined KE approaches included research evidence and clinical guidelines and clinical/epidemiological/surveillance data as well as the exchange of research/practitioner/policy knowledge. No studies reported on the inclusion of lay knowledge, however, two studies described the inclusion of informal sources of knowledge available from blogs, list servers and social media [37,38]. Only one study adopting a combined KE approach referenced an underpinning theoretical framework, which was situated in the transfer of evidence into practice [54].

Global level activities included the development of a KE strategy for African countries [54] and networks and processes to exchange and disseminate surveillance data and expertise between countries [37]. Organisational level activities included internet-based web resources and a social media platform designed to rapidly disseminate and exchange knowledge about Covid-19 [57] as well as the deployment of public health epidemiologists acting as links between surveillance data and public health staff [45]. Specifically at the preparedness stage, Brookes developed a conceptual model describing flows and synthesis of information to disseminate and exchange knowledge relevant to planning for infectious diseases [38].

### Environmental crises

Two studies reported on evidence access mechanisms during environmental crises; these were used during recovery and mitigation/preparedness stages [75,87]. Activities concerned the dissemination of guidance to professionals planning for extreme weather events [87] and local builders involved in housing construction [75].

A larger proportion of environmental crisis studies (n = 14) reported on active KE [55,67–69,71,73,74,76,77,79,81,84,85,109]. Theoretical frameworks underpinning active KE in environmental settings were aligned with participatory and collaborative principles, aiming to develop a more holistic understanding of a crisis inclusive of a range of knowledge [67–69,71,74,77,81,83]. Active KE environmental crisis approaches encompassed the exchange of formalised knowledge (e.g. good practice recommendations, technical guidance), [75,78,83,85,86] organisational and policy/professional knowledge from different sectors and disciplines (e.g. water managers, NGOs and international agencies), [67,69,71,73,74,77,81–85] the knowledge of volunteers following a crisis such as information about the location of damage or weather conditions [71,76] as well as local contextual knowledge of communities living in affected areas [73,74,77,79,81–85]. The use of scientific data/research and academic knowledge was referenced explicitly in a small number of studies [67,69,81,83,85].

Active KE during crises was reported at all stages with several studies highlighting that the KE was relevant to more than one crisis stage. Where described, distinctions between KE approaches at different stages are highlighted below. At the preparedness and mitigation stages, for example, the use of multi-component KE (e.g. workshops, drama, exchange programmes, partnerships, participatory action models) informed collective efforts to reduce the risk of environmental crises in local systems [68,79,81,82]. As part of mitigation activities to reduce the risk of natural hazards, a systems approach to KE utilised co-design, knowledge production and collaborative planning with research organisations, public and private-sector representatives, local NGOs and conservation groups [83]. Similarly, a conceptual simulation model of a fresh water management system and stakeholder platform aimed to bring together practitioner and scientific data to address policy issues in the context of drought and low river-flows [69]. In the immediate aftermath of crises that involved a risk to life, ICT networks and internet social networking was used as part of KE during response efforts, including, for example, the sharing of data by local residents with agencies such as reports/visuals on location of damage or people requiring assistance [71,76].

During the recovery stage, three studies reported on projects to encourage safer construction practices in localities affected by weather related events and earthquakes involving KE delivered through NGOs or donor programmes [73,74,77]. In these studies, the involvement of communities of place often aimed to encourage the uptake of existing good practice guidance as part of housing construction. One different approach involved a workshop and network with the Sri Lankan Tamil community who co-designed distress relief models to promote recovery and support community needs in both local and international contexts [85].

All environmental studies (n = 7) reporting combined KE took place during the response stage, with three studies also highlighting the relevance of KE for preparedness [78,88,102] and recovery stages [70,86,102]. Four studies reported the relevance of KE for the mitigation stage [70,72,78,80,102]. As with active KE, there was a prominent focus on multi-directional KE combining diverse knowledge types. At a global level, for example, a communities of practice with multi-component KE activities was established to address an identified KE gap in climate change and health [72]. At a local level, organisational structures for KE during an earthquake’s response and recovery stages included the use of knowledge hubs/web portals combined with forums and informal networks [70] and the development of interactive platforms to enable professionals to access and learn from each other’s disaster prevention plans [80].

### Technical crises

Two studies reported on access mechanisms [90] and combined approaches to KE [91] at preparedness and response stages. Theoretical frameworks underpinning KE were not described. Evidence access mechanisms aimed to improve awareness of potential threats related to nuclear hazards and radiological emergencies through the development of repositories [90,91]. One of these studies also included a multi-stakeholder project to address gaps in nuclear and radiation preparedness [91].

### Terror related crises

Two studies reported on evidence access mechanisms and combined approaches to KE [91] at preparedness [93] and recovery stages [92]. Theoretical frameworks underpinning KE were not described. Evidence access mechanisms aimed to improve awareness of potential threats through the development of repositories collating bio-terrorism related information [93]. In the context of recovery, a university–community KE programme was designed to tackle widespread poverty and unemployment in regions with histories of terror related conflicts [92]. In this study, Nigerian academics received mentorship from UK-based knowledge transfer experts and then delivered an entrepreneurship curriculum and organised outreach placements for students, which enabled experiential and academic knowledge to be brought together.

### Humanitarian crises

Two studies on humanitarian crises reported the use of evidence access [89] and combined approaches to KE [26]. Theoretical frameworks underpinning KE referenced knowledge management, sharing and retrieval in organisational contexts [26,89]. For example, a review of KE within peace keeping missions resulted in the establishment of a revised organisational KE structure for the United Nations including dedicated KE professionals, knowledge management systems, an online ‘communities of practice’ and the translation of knowledge into institutional policies and procedures [26].

### Various crisis types

Studies reporting on more than one crisis type did not compare whether approaches to KE differed in different contexts or crisis stages. Theoretical frameworks were more likely to be mentioned in the context of active exchange and combined approaches. In some studies, knowledge management, transfer, translation or exchange was interpreted as a process through which knowledge was shared, exchanged and utilised to inform learning for policy and practice [24,96,97,105,107,108]. Additional studies placed an emphasis on the role of networks and multi-directional KE [111] and principles of community-based participatory research [114].

Evidence access mechanisms (n = 8) included websites and repositories [94,113]; organisational knowledge management systems [104]; data platforms [95,101,109] and the role of professionals with a knowledge translation function [98,100]. Research evidence and data were the primary type of knowledge accessed. Active KE (n = 8) covered a range of knowledge, reflecting those outlined under crisis types above. However, exchange involving lay knowledge was evident to a lesser degree. Active KE included workshops and reflective exercises [96,97,110], interactive tools [99] and participatory approaches [111,114]. The co-location of professionals from different sectors [103] and use of placements [111] was also described. Combined KE approaches (n = 5) included organisational structures for the management and exchange of knowledge [108,112], a website to disseminate systematic review evidence combined with a communities of practice [105] and a global network to improve access to and exchange of knowledge on nutrition information [106]. One review mapped a range of different approaches to translating evidence in different organisational contexts which included the use of stakeholder dialogues and rapid evidence service, dissemination activities, evidence websites and skill development programmes [24].

### Facilitators and barriers to KE

Fifty seven studies reported on facilitators and sixty six studies reported on barriers to KE in crisis settings. Below, our synthesis firstly presents the barriers or facilitators to KE that were reported to be associated with particular crisis stages or crisis types (e.g. the emergency nature of a crisis). We then summarise barriers and facilitators associated with KE identified in studies more generally. These latter factors (for example, a lack of resource for KE in organisations) were not reported as specific to crises types/stages but nevertheless were perceived to amplify or facilitate the success of KE during crisis contexts.

#### Barriers to KE

The emergency nature of some crises, where there was a short timescale to act due to life threatening circumstances affected the feasibility of KE [57,98]. Similarly, digital inequalities (e.g. the lack of internet in remote areas) [26,71,76,98] or disruption to information infrastructure following a crisis [76,98] had a bearing on the exchange of knowledge between key actors (for example, the location of disaster victims or extent of local damage). Yahya and colleagues [88] observed one complexity of KE in environmental crises, such as flooding, is that there is significant uncertainty and unpredictability about the disaster’s scale and reach. Finally, two studies referred to the need for robust evidence to inform KE in increasingly complex and frequent crises situations, but noted this was constrained by the practical challenges of conducting research and evaluating models of KE in emergency situations [26,96].

During the response stage of a crisis, barriers were identified related to the application and availability of research evidence. A lack of timeliness of research evidence [32,41,43,44,48,54,58,100,108] and difficulties in accessing information rapidly [24,54,57,58,60,74,86,93,94,102] were reported to impede decision making. Related to this, academic and commercial competing interests, such as the reluctance to share data before publication [37,51,54,69,70], were noted to affect access to knowledge in a small number of studies. Even where research evidence was available, challenges were reported in using this for decision making [24,38,41,43,57,58,67,78,99,109,110,113], with gaps in research and knowledge translation ‘literacy’ among both researchers and professionals affecting whether and how evidence was used [43,45,47,51,54,67,72,86,113]. Time pressures faced by policy makers and professionals also affected their capacity to access and use research evidence [42,53,104,108,109]. As a result, one paper reported on the selective use of research evidence [44]. Also during the response stage, concerns about reliability and trustworthiness of knowledge were reported. These included concerns about misuse of knowledge [68,71], miscommunication and fake news [24,44,71,76] and more general concerns about credibility and quality [48,50,53,86,109]. Where crises required an interagency response, relational factors (e.g. a lack of trust) [24,31,41–44,47,70,78,89,96,102,107,110] or technical barriers [32,52,70,87,103,107,110] affected a willingness and ability to share knowledge across sectors and organisations. Similarly at a global level, challenges in the coordination and sharing of knowledge were reported when crises such as pandemics affected multiple countries [48,61,66,72,89].

At the recovery stage, barriers in community settings were particularly evident in the context of environmental crises where KE sought to engage residents or community groups as part of planning and rebuilding local infrastructure (e.g. housing). A lack of financial resources to implement changes or technical ‘know how’ could limit a community’s ability to implement ‘best practice’ guidance such as safe housing construction [77,82,102,114]. While not specific to the recovery stage, a lack of sensitivity by organisations to local cultures and contexts [70,74,98,102], literacy and language issues [74] and a lack of trust between agencies and communities [114], also affected the success of KE in this setting.

Preparedness or mitigation planning could be hampered by a loss of institutional knowledge when the tacit knowledge of crisis practitioners was not shared from one crisis to another [26,66,70,73,86,100,102,104,108,112]. High levels of staff turnover, particularly in NGOs, was a key factor influencing this [26,61,70,73,104,108,112]. One study highlighted that the infrequency of particular types of crises, such as tsunamis, also meant knowledge could get lost from one crisis to the next [81].

Finally, several studies noted general barriers associated with KE. Practical barriers were associated with maintaining information systems for KE (e.g. platforms/databases) [32,34,40,47,56,60,64,65,76,86,93,102,103,109,112], a lack of IT/technical capability [32,71,84,107,110] and sensitivities with sharing data [52,76,102,103]. Barriers associated with delivering KE were also highlighted (e.g., insufficient reach of activities) [26,53,54,74,106,112], with several studies pointing to a lack of human and financial resources to support KE [34,47,53,54,57–59,65,66,72,74,75,81,92,102,106].

Barriers related to macrolevel factors were usually referred to in general terms (e.g. political, economic, geographical, social factors) [24,26,34,37,38,44,48,53,66,71,78,92], without always providing detail of how these factors affected the crisis at different stages. One exception was the impact of conflicting public health and economic priorities which could impede political willingness to publicly disclose knowledge because of the potential impact on a country’s economy and tourist industry [26,37,44].

#### Facilitators to KE

During the response stage, where a country’s infrastructure was significantly damaged during an environmental crisis, the use of informal networks among communities and workers was critical in facilitating flows of knowledge [71,76].

Several factors were also reported to help improve accessibility of research evidence and data, as well as support its use. These factors were connected to the response stage of a crisis and were largely reported in the context of pandemics. Facilitators included removal of financial barriers, such as journal fees [24,40,49,58,60,62,63,93], dissemination and evidence access mechanisms (e.g. weekly reports, repositories and platforms) [37,50,58,64,113]; and the provision of materials in multiple languages [64]. Researchers’ credibility and neutrality also increased policy and practitioners’ confidence in using evidence [40,43,44,62,88,98].

During crises spanning organisational and country boundaries, the existence of pre-existing networks [35,70,78,102,103,107] provided mechanisms for flows of knowledge. Informal networks also facilitated a more rapid sharing of knowledge, compared to formal structures [35]. Trust and willingness to share knowledge between sectors and professionals [46,78,103,104] and clarity about roles and responsibilities [85] also facilitated sharing of knowledge. Similarly, at global level, openness between country stakeholders [37] and as intercountry networks and collaborations [37,38,46,49,72,102] promoted knowledge exchange.

With the exception of studies of the Ebola pandemic [53,61], the majority of studies reporting facilitators in community settings were in the context of environmental crises [68,69,74,78,82,84,85]. These facilitators were generally not specific to a crisis stage. Factors enabling local communities to participate in KE processes included the existence of structures for participation and the involvement of community leaders [61,68,78,82], alongside approaches that built trust and mutual respect [74,85]. In one study, personal experience of crises was reported to motivate the public to get involved in KE preparedness and mitigation activities. Where lay knowledge was included [53,69,84,103,111], it was reported to increase the likelihood of crisis responses being effective and appropriate, with sensitivity to cultural and local contexts also affecting meaningful community participation and appropriate crisis responses [61,64,74].

More generally, a number of factors were identified as enabling KE that were not specific to a crisis type or stage. These factors included the employment of a professional to facilitate and promote KE [26,77,102,106,112] and ensuring KE activities were responsive, adaptable and proactive [44,46,64], relevant and credible [53,61]. as well as opportunities for researchers to develop new skills in KE where this was new to them [61,81]. Similarly, existing capacity for KE (staffing and resources) in organisations [44,72,104,106] and the ability to mobilise in-kind time and expertise [72] in response to a crisis was highlighted as important for effective KE. Technology was reported to facilitate stakeholder participation and reach [56], augmenting more traditional methods of dissemination across remote or multiple locations [72] and reducing travel and geographical barriers [57,72]. Within organisations, leadership and management commitments [24,26,31,53,70,81,102,104,107,112] created an enabling environment for KE. Having functioning information systems and infrastructures (or being able to quickly mobilise these) [44,65,70,76,88,106,109,112] and use of effective IT [24,59,66,67,80] also facilitated the rapid exchange of data and other knowledge when a crisis occurred. Few studies referred to macro-level factors as facilitators of KE. Where this was described, studies referred to country-level political and legal contexts, such as the extent of political freedom and a population’s access to the internet [40,107].

### KE impacts and recommendations

#### KE impacts

Limited evaluation of the impact of KE activities was reported and where impacts were described, they tended to be rooted in either participant or researcher perspectives, but not formally measured through evaluative data collection methods. The relatively small number of studies that did formally evaluate the impact of KE activities utilised a range of data collection methods, including participant observation, interviews [48,70,75,78,79,83] and surveys [42,77,81,82,84,96]. In addition, a number of studies of evidence access models of KE reported output indicators which measured the reach of KE resources and activities (e.g. number of subscribers, geographical spread of knowledge users), and less frequently, the satisfaction with resources [26,40,49,57,58,60,63–65,109]. In one study, health departments reported high levels of information dissemination, but targeted professionals reported much lower receipt of the same information [42]. The researchers suggested this showed that professionals may find the information redundant since they receive or seek out the same information from other trusted sources [42].

KE was reported to have contributed to improving intersectoral relationships, communication structures and understanding of roles and opportunities, as well as sharing of experiences, thereby empowering organisations to more effectively prepare for and respond to crises [41,44,48,70,83,96,97,103,112]. For example, one study described how the co-location of two agencies led to improved information-sharing practices and the ability to identify risks utilising fewer resources [103]. Several studies also described how KE efforts improved connections between different stakeholders within a system, including between academics and practitioners and local communities [58,85,112]. One study also reported trust reducing between organisations as a consequence of KE [70]. One study reported that KE activities expanded action beyond those immediately engaged in the KE activity; in this study, resources developed by a community of practice influenced Canada’s strategic global health research priorities and the community of practice was subsequently expanded internationally, thereby strengthening research collaborations between the Global North and Global South [72].

At an organisational level, it was argued that KE led to lesson learning about crisis responses (e.g. capabilities, technical and staffing issues) [96,97] and the improvement of flows of knowledge within organisations [26,112]. This contributed to institutional changes in future preparedness and response strategies [96,97]. In two studies, the implementation of KE activities also extended opportunities for learning and training for students [72,95].

Access to evidence-based resources was reported to facilitate the widespread dissemination of evidence and to inform the development of crisis responses [50,87,94,98]. In addition, rapid and timely access to knowledge was reported to support more accurate and informed decision making during immediate crisis responses [37,46,59,103]. Some studies reported that effective sharing of evidence and data on emerging outbreaks, and accessing and applying relevant knowledge led to intervention efforts which were able to curb the spread of infectious diseases [40,54,55,60]. Where KE concerned the sharing of data and intelligence, this was reported to have improved efficiencies in data requests and built momentum for a data sharing agenda [51,70], increased the use of information by professionals and decreased the amount of time spent summarising data [45]. The sharing of knowledge also informed the development and testing of KE models or tools which were reported to effectively combine scientific and local knowledge [43,69,83], and be acceptable to users [36,108,109].

Within local communities, studies reported that KE led to the development of specific skill and knowledge sets, such as the development of business skills that could be utilised within the context of an on-going conflict [92], knowledge about how to respond to a crisis [81] or building construction skills and knowledge for post-disaster re-building, retrofitting or mitigation [75,77,79,82]. However, two studies reported that while participants had higher levels of knowledge about earthquakes and risk reduction, that this did not translate into skills for retrofitting buildings to make them safer, or that residents largely continued to live in homes unsafe in the event of future earthquakes where material resources were not available [82,84]. Another study highlighted that while an owner-driven approach to housing reconstruction was associated with higher levels of satisfaction with respect to influencing building design, participants perceived donor-driven housing reconstruction to be of higher quality and more durable [77]. Several studies also underscored the importance of sharing and integrating lay knowledge into crisis planning or directing a crisis response [53,72,76]; for example, one study described how using information from micro-blogs enabled responders to rescue trapped individuals following a typhoon when official reporting systems became overloaded [76].

Some authors reported that adopting a participatory approach that actively involved community members was key to ensuring programme sustainability and adaptability [68,78,83]. In two studies, authors described this approach as a key reason for a shift in strategy away from response to mitigation and adaptation approaches [68,83]. In another study, water management structures that were participatory and inclusive of both technical and local knowledge were more adaptive and responsive to water management crises [78]. However, the authors note variability across different organisational structures, with some participation from members of civil society waning during an immediate crisis response [78].

#### KE recommendations

The KE recommendations put forward in included studies ranged from those that were project- and context-dependent to more general recommendations for enhancing KE processes and outcomes. Authors recommended providing timely and relevant data in order to facilitate effective crisis responses [45,51,64]. Technical recommendations for KE included integrating clinical and data management systems, the use of web portals, information technology and platforms to improve access to knowledge [26,31,37,42,44,48,52,54,56,65,70,76,102,109,110,112]. Some authors underscored the need for these systems to be curated and managed by knowledge brokers with relevant crisis expertise [48,70,98,112], as well as be user-friendly and straight-forward, something that could be achieved through the active involvement of end-users in the design and piloting of tools [65,67,108]. In a similar vein, many authors recommended participatory or community engagement approaches that incorporate and exchange knowledge from a range of stakeholders to ensure knowledge generated and information-sharing practices address the needs of end-users or local communities [43,49,65,69,71,72,74,77,81,85,113].

KE roles and systems were suggested to facilitate more active exchange by nurturing relationships and interactions within and across organisations, and amongst different actors and sectors [26,31,41,43,54,70,76,97,102,112]. Authors suggested that incentives and appropriate resources, governance and senior leadership commitment are needed to encourage data and knowledge sharing [48,51,53,73,108,112]. To facilitate the exchange of knowledge, study authors recommended strengthening both informal and formal networks for information sharing and the need for researchers and practitioners to better understand the context and cultures in which KE occurs [31,33,41,42,48,71,73,92,102,113]. This was highlighted as particularly important in resource-poor settings where capacity may be an issue [48,71]. Where resources were developed, there was a need for training for those intended to use the resources [103,108].

Some studies made specific recommendations to capture ‘lessons learned’ from previous crises to inform future mitigation, preparedness and response efforts in a form of two-way KE [73,102,110]. Other studies made recommendations for knowledge transfer that described how researchers can better disseminate their work and suggested that results need to be actionable and presented in such a manner that make them usable to non-technical stakeholders. In addition, some authors recommended that researchers should carefully consider open access publishing options to ensure evidence is available to those in middle- and low-income settings [41,60,108,113]. Another recommendation was that grant proposals include KE as a specific budget item, including both training for researchers in KE and funding of KE processes [61].

Finally, the need for organisations to be continually adaptive and innovative in their approach to KE was also recommended, alongside the need to evaluate KE approaches to provide evidence about the impact of KE strategies on crisis preparedness and response [26,48,68,74,81,90].

## Discussion

### Key findings and comparison with broader literature

This scoping review sought to identify and compare models and impacts of KE conducted in different types of crisis settings. The majority of identified studies involved the exchange of research evidence and data, as well as practitioner and policy knowledge. Approximately a third of the studies involved the exchange of lay knowledge. In this body of literature, KE efforts focused on the active exchange of knowledge, as well as efforts to improve the accessibility of research evidence and data. While the identified studies were all relevant to the social determinants of health, the majority did not explicitly discuss or analyse these determinants in their findings or make explicit reference to health or health inequalities.

Most studies identified in the review were concerned with pandemic or environmental crises and therefore much of this section will compare knowledge exchange conducted within these types of crises. Pandemic papers centred on a range of different types of outbreaks, with many recent papers focusing on Covid-19 [44,46,49,50,52,57,58]. KE during environmental crises was more commonly associated with specific events (e.g. an earthquake or water-related emergency), rather than sustained crises such as climate change, global poverty and health inequalities [72,78]. Our review identified fewer studies exploring KE during technical, terror-related or humanitarian crises. This may partially reflect the relative infrequency of certain types of crises but is unlikely to explain the limited number of humanitarian crises identified. In comparison, a recent review on the use of research evidence for decision-making in crisis zones in low- and middle-income settings similarly identified many studies of environmental crises, but more conflict-related (humanitarian) crises than our searches [24]. Their review also found fewer pandemic studies, which may suggest an evidence gap in pandemic-related KE studies conducted in low- and middle-income countries.

We identified studies conducted across all crisis stages. Some studies allowed us to compare how KE is conducted at different stages of a crisis enabling us to draw out possible differences in the purpose of KE at these different stages. However, as will be discussed below, there were limitations in the extent to which we were able to compare models of KE between crisis stages and types.

While the majority of pandemic studies reporting on KE did not explicitly address the social determinants of health, the identified studies on KE during environmental crises provided evidence that related directly to supporting action on social determinants. This was largely due to the nature of these types of crises; for example, KE processes to support post-emergency housing reconstruction programmes featured strongly in the identified literature. KE within pandemic contexts largely focused on the immediate public health response to tackling outbreaks in populations rather than clearly addressing social and economic consequences of the outbreak (e.g. social isolation, financial hardship). Moreover, across all types of crises, there was little evidence of KE as part of longer-term recovery from the social and economic impacts of crises. The only exception to this was an entrepreneurship programme developed to support recovery from terror-related conflict in a sub-Saharan African setting [92].

Notable differences were observed in KE models particularly when environmental and pandemic crises were compared. KE during pandemics adopted a more linear flow of research evidence, data and formalised knowledge to support policy and practice decision making. Where exchange occurred, this mainly spanned organisational boundaries and networks as part of official responses to outbreaks. It also sometimes involved interaction between researchers and policy makers/professionals in an effort to address the research to practice ‘gap’ [116]. In contrast, KE during environmental crises often favoured more collaborative processes of knowledge production. KE approaches during environmental crises were more likely to highlight the need for a plurality of knowledge to understand and respond to a problem, including lived experience of the public [117]. This finding may reflect differences in underlying epistemological beliefs across disciplines which shape the nature of KE [118]. For example, a review by Fazey and colleagues [18] identified that KE in health settings was largely driven by a more positivist position involving a focus on the dissemination and translation of evidence compared with KE in environmental settings which was more aligned with a relativist view that emphasises the co-creation of knowledge.

As noted above, environmental crises were more likely to include lay knowledge as part of the exchange. Another reason for this may concern the specific nature of environmental crises, whereby, during the immediate response, community involvement is essential to support emergency efforts [119]. During the recovery period of environmental crises, KE models were sometimes more reflective of a one-way flow of knowledge instigated by organisations (often NGOs or donors) to encourage the implementation of safer building construction programmes in communities [74]. Overall, however, there was recognition of the need for lay knowledge to inform programmes which were perceived as historically insufficiently sensitive to local context in the past [73,77].

In most pandemic studies we reviewed, lay knowledge was largely absent from the KE model. Notably, this was not the case for studies of KE during the Ebola outbreak [53,55,61]. Indeed, anthropological literature on Ebola outbreaks in West Africa underscores the ways in which the knowledge and response strategies of local populations have been adopted, replicated, and scaled-up [120–122]. Richards describes how a ‘people’s science’ [121] grounded in locally-developed practice in conjunction with the international response formed knowledge co-production processes that were able to effectively reduce transmission of the virus [121]. Our findings also resonate with those of a review of community engagement in pandemic responses, where community engagement strategies and practices were found to be less developed in higher income country settings, compared to low and middle-income contexts [123]. These findings suggest that cultural, economic, geographical and historical factors may play a role in creating a culture of lay knowledge use in low income country contexts and would warrant further investigation.

Writing in the context of climate change in 2010, Jasanoff has challenged the notion of ‘legitimate knowledge’ (i.e. science and evidence), calling for knowledge production and exchange processes to involves ‘an immense variety of actors including the local and translocal’ [124 p.249]. This perspective also builds on Jasanoff’s (and others’) work that explores how the failure of science to engage with local knowledge and experience has led to significant failings in crisis responses in past decades [125]. Where KE was led by public health organisations, the concept of legitimate knowledge continued to be perceived (albeit implicitly) as largely belonging to the practitioner, policy maker and researcher. Notwithstanding this, there were some exceptions that offer learning for public health, notably, a community of practice on climate change and public health [72].

While we excluded studies that solely concerned a one-way mode of risk communication to the public during a crisis, some of the included studies involved a discussion of risk alongside formalised KE efforts. The studies we identified that reported on the involvement of local communities as part of the crisis response, in particular, shed some light on how risk is communicated to, and with, the public. In these studies, the community was not a ‘passive’ recipient of knowledge, rather an active recipient, using and sharing knowledge to inform the rescue or response efforts. Okada and Matsuda, writing about developing risk communication strategies to improve preparedness for disasters make a similar point, arguing that during a crisis the ‘roles of risk experts are shared by citizens, nonprofit organizations and researchers in order to deal with a problem with much uncertainty’ [126 p.640]. Covello and colleagues argue that while risk communication should be a two-way, collaborative and interactive process between experts and the public, that risk communication is often one-way and hindered by a lack of trust and coordination [127]. Renn, writing about risk communication strategies argues similarly that ‘stakeholder involvement and public participation in risk assessment and management process help to improve the quality of decision making and […] avoid damaging and time-consuming confrontations later on in the decision-making process’ [128 p.91]. These arguments echo findings from this review on trust, collaboration and the production and use of lay knowledge. Taken together, this suggests further research might usefully compare these theoretically overlapping bodies of literature.

With the exception of some aspects of crises (e.g. their emergency nature), many factors influencing KE–such as the importance of organisational cultures supportive of KE–were not unique to crisis settings although some factors were more evident at particular crisis stages. This included, for example, the loss of institutional knowledge impacting on preparedness or mitigation against future crises. Similar to other reviews of KE, factors influencing the use of research evidence, including its timeliness and salience to decision making, were noted by studies included in this review [24,129,130]. Nevertheless, these challenges are likely to be amplified during crisis situations, for example, where compressed timescales affect the sources of knowledge used to inform decision making or whether a lack of trust has an impact on the extent to which vital information is shared between organisations or countries.

### Strengths, limitations and challenges

The review focused on a broad range of conceptualisations of KE and types of knowledge. This created challenges for synthesising such a disparate body of literature rooted in a range of contexts, including global, national, local, inter- and intra-organisational and community settings. Nevertheless, this broad conceptualisation also allowed us to surface the ways in which a range of different forms of knowledge are used during crises.

In order to conduct the review, we needed to operationalise the concepts relevant to our research questions and place boundaries on the scope of the literature we reviewed. We utilised our research aims and perspective to make choices about the search terms and inclusion and exclusion criteria. Other research teams might have chosen different boundaries, for example, using different search terms that identified additional types of crises or those that incorporated different types of evidence sources [131]. One challenge was operationalising a distinction between risk communication and knowledge exchange, recognising that the two can overlap substantially.

We identified a relatively small number of technical, terror-related and humanitarian crisis studies. This is perhaps a limitation of our search strategy or may reflect a general gap in the peer-reviewed evidence-base where many crisis responses are led by NGOs and findings may not be published in academic journals.

In over half of the identified studies, KE processes were considered at more than one crisis stage and authors did not necessarily discern if, how, or to what extent, the KE activities were adapted to different stages. In addition, the term ‘response’ was often used in studies as an all-encompassing term to refer to any stage of disaster management. This could make it challenging to disentangle KE at specific crisis stages. This blurring of categories may, of course, also reflect the nature of crises themselves, which do not have a linear trajectory or fall neatly into stages; the Covid-19 pandemic with its overlapping waves demonstrates this.

## Conclusions: Implications for research and practice

This review highlights a number of lessons for future KE and research. First, the evidence on KE during crises is predominately generated through descriptive case studies. We identified relatively few studies which have undertaken robust evaluation of KE processes and their effectiveness. There is, therefore, a need to develop robust methods for conducting and evaluating KE during crises, given that such research presents a range of ethical and practice challenges [132].

Second and related, KE efforts need to be designed in a manner that appropriately reflects the complexity inherent in crises, including their non-linear trajectories that are characterised by uncertainty and unpredictability [26,83]. In crisis settings, decision making, including a calculation about risks, uses a range of sources of knowledge that are created within a complex social system of interactions among multiple stakeholders [133–135]. Such a framing suggests there may be utility in explicitly applying a complex systems lens to KE in crisis settings. While we identified a small number of studies that used systems approaches to understand and deliver KE [35,55,83], these were in the minority. The adoption of a complex systems framing and the utilisation of systems or complexity-informed methods to design and evaluate KE may offer opportunities to utilise research methodologies that foreground the complex systems in which crises occur, account for unpredictability and are adaptive to emergent findings [136–138]. This approach could also be helpful in considering opportunities for more holistic responses in designing KE processes to support future, overlapping crises, as well as the need to develop long-term KE strategies that exist beyond immediate crisis responses.

Third, there was considerable variation in the extent that lived experience of communities was valued and included as part of the KE, highlighting the need for testing and evaluation of KE models that facilitate exchange between professionals, lay stakeholders and researchers at all stages of crises. This holds particularly true for high-income countries.

Fourth, the relative lack of focus on KE as part of programmes to address living and working conditions indicates a need for a more upstream focus in planning broader recovery from crises such as Covid-19, particularly given what is known about inequalities experienced by different populations during and after crises.

Finally, given the increasingly multiple and overlapping nature of crises (e.g. climate change, pandemics, flooding) recovery from the Covid-19 pandemic and other crisis will require a focus that goes beyond ‘returning to normal’ in order to address the ways in which our public systems and environments shape the public’s health.

## Supporting information

S1 Checklist PRISMA checklist. (DOCX)

S1 File Example search strategy: Medline. (DOCX)
