# Supplementary material for: Knowledge exchange in crisis settings: A scoping review
Source: PLoS One. 2023 Feb 24;18(2):e0282080. doi: 10.1371/journal.pone.0282080 (PMC9956070; doi:10.1371/journal.pone.0282080)
Supplement: S1 File — (DOCX) [file pone.0282080.s002.docx]

# **S2. Example search strategy: Medline**

1 AND 2 AND 3

1) Cris#s OR disaster* OR emergency OR emergencies OR war OR wars OR conflict* OR forced displacement OR recovery planning OR pandemic* OR Ebola OR Covid OR lockdown* OR austerity OR financial crash* OR recession* OR Armed conflicts/ OR Emergencies/ OR Disasters/ OR Natural Disasters/ OR Pandemics/ OR Economic Recession/ Limit to English language

2) Public health OR population health OR community health OR mental health OR well-being OR wellbeing OR health promotion OR health inequalit* OR health inequit* OR social determinant* OR upstream determinant* OR place-based OR housing OR transport* OR food OR nutrition OR water OR sanitation OR crime OR violence OR community safety OR school* OR education OR community development OR community empowerment OR Public health/ OR Population Health/ OR Mental health/ OR Health promotion/ OR Social Determinants of Health/ OR Housing/ OR Food/ OR Water/ OR Sanitation/ OR Crime/ OR Violence/ OR Schools/ OR Education/ Limit to English language

3) Knowledge exchange OR knowledge transfer OR knowledge translation OR knowledge dissemination OR knowledge diffusion OR knowledge utili#ation OR knowledge management OR knowledge adoption OR knowledge uptake OR evidence utili#ation OR “evidence use” OR “knowledge use” OR Knowledge management/ Limit to English language
